# A Liquid Metal Balloon for the Exfoliation of an Ultrathin and Uniform Gallium Oxide Layer

**DOI:** 10.3390/molecules29245894

**Published:** 2024-12-13

**Authors:** Anar Zhexembekova, Seongyeop Lim, Cheongha Lee, Yun-Tae Kim, Chang Young Lee

**Affiliations:** 1 School of Energy and Chemical Engineering, Ulsan National Institute of Science and Technology (UNIST), Ulsan 44919, Republic of Korea; anar201@unist.ac.kr (A.Z.); lsy981202@unist.ac.kr (S.L.); cheongha@unist.ac.kr (C.L.); ytkim@unist.ac.kr (Y.-T.K.); 2 Graduate School of Carbon Neutrality, Ulsan National Institute of Science and Technology (UNIST), Ulsan 44919, Republic of Korea

**Keywords:** liquid metal, gallium oxide, balloon, exfoliation, carbon nanotubes, gas sensor

## Abstract

We report the exfoliation of ultrathin gallium oxide (Ga_2_O_3_) films from liquid metal balloons, formed by injecting air into droplets of eutectic gallium–indium alloy (eGaIn). These Ga_2_O_3_ films enable the selective adsorption of carbon nanotubes (CNTs) dispersed in water, resulting in the formation of a dense, percolating CNT network on their surface. The self-assembled CNT network on Ga_2_O_3_ provides a versatile platform for device fabrication. As an example application, we fabricated a chemiresistive gas sensor for detecting simulants of chemical warfare agents (CWAs), including diisopropyl methylphosphonate (DIMP), dimethyl methylphosphonate (DMMP), and triethyl phosphate (TEP). The sensor exhibited reversible responses, high sensitivity, and low limits of detection (13 ppb for DIMP, 28 ppb for DMMP, and 53 ppb for TEP). These findings highlight the potential of Ga_2_O_3_ films derived from liquid metal balloons for integrating CNTs into functional electronic devices.

## 1. Introduction

Gallium-based liquid metal has been highlighted as a promising electronic material for flexible and stretchable devices thanks to its high conductivity and fluidic properties [1]. One of the unique properties of such liquid metal is the spontaneous formation of an ultrathin gallium oxide layer on the exterior upon exposure to air. Numerous studies have utilized the oxide skin as a mechanical support that helps maintain the 3D shape of the liquid metal after deformation [2,3]. Moreover, the oxide skin of the gallium-based liquid metal offers a simple way to create two-dimensional (2D) metal oxides at room temperature [4,5]. The oxide skin can easily be separated from the underlying liquid metal due to their weak attachment, similar to peeling off layers in 2D materials like graphene [6,7]. Thus, for further application of the gallium oxide layer, it is important to devise a straightforward approach for peeling off the gallium oxide layer, while minimizing its damage.

Previous studies have demonstrated various methods for separating the gallium oxide, Ga_2_O_3_, from the gallium-based liquid metal alloys by contact-printing the liquid metal droplet on a substrate [4,8,9], squeezing the droplet across a substrate [10,11,12], or pressing it between two surfaces [13,14]. The obtained ultrathin gallium oxide films have been successfully used as an insulating material in electronic devices [15] and as a protective coating for materials such as WS_2_ [8] and graphene [16] against damage during subsequent processing steps. However, these approaches often suffer from non-uniform oxide layers and undesired metal residues [17,18]. While a more controlled method using air bubbles in water was reported [4], the requirement for an aqueous environment has limited the practical applications of the approach.

In this work, we demonstrate the exfoliation of an ultrathin and highly uniform gallium oxide film from a liquid metal balloon. By injecting air into a liquid metal droplet, we were able to inflate the droplet and transform it into a liquid metal balloon, during which the oxide layer was separated from the bulk liquid to form a translucent film. The balloon was then transferred onto a silicon substrate by simple contact-printing, which resulted in a gallium oxide layer with uniform thickness and smooth surfaces. Surprisingly, the gallium oxide film prepared in this manner facilitated the selective adsorption of carbon nanotubes (CNTs) dispersed in an aqueous solution. By patterning electrodes on the percolating network of CNTs, we fabricated chemiresistive gas sensors for the detection of various toxic chemical compounds. Hence, the results suggest that the exfoliation of the gallium oxide layer from a liquid metal balloon and the subsequent deposition of CNTs on the oxide can serve as a unique approach for potential applications in various electronic devices.

## 2. Results and Discussion

**Figure 1:**
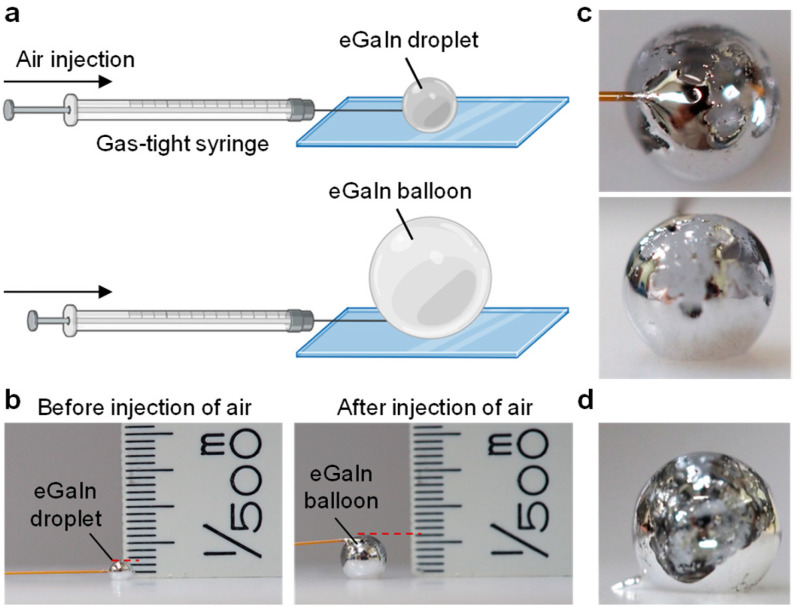
Preparation of a liquid metal balloon. (**a**) Schematic illustration of the liquid metal balloon formation by injecting air into a liquid metal droplet using a gas-tight syringe. (**b**) Optical images of an eGaIn droplet before (**left**) and after (**right**) air injection, showing a diameter increase from 1 mm to 2.4 mm. (**c**) Optical images of the liquid metal balloon collected from two different angles. (**d**) Optical image of the liquid metal balloon after the needle was removed.

A liquid metal balloon was created by injecting air into a droplet of eutectic gallium-indium alloy (eGaIn) using a gas-tight syringe, as illustrated in Figure 1a. The flow rate was controlled at 1 mL/h by a syringe pump. The eGaIn droplet being infused with air initially showed little change in size, while its surface gradually became tighter. However, after a few minutes of air injection, the droplet rapidly expanded into a balloon. A ruler was placed next to the droplet to measure the size before and after the air injection (Figure 1b). The height increased from 1 mm for the eGaIn droplet to 2.4 mm for the balloon, which corresponded to a ~14× volume increase from 0.52 μL to 7.2 μL. 

The droplet-to-balloon transformation was enabled by the ~3 nm-thick gallium oxide skin [4,19], which formed spontaneously on both the exterior and interior of the liquid metal when exposed to air. During the balloon formation, the oxide layer was spontaneously separated from the liquid metal. The formation of the liquid metal balloon led to the spontaneous separation of the oxide layer from the liquid metal surface, resulting in partially translucent regions in the balloon (Figure 1c). The translucent film and the balloon structure remained stable, showing no deflation or shrinkage, even after the air injection stopped and the needle was removed (Figure 1d). The observed delamination of the liquid metal from its oxide layer is attributed to their weak interfacial bonding [7] and to the electron density distribution being minimized at the liquid metal and oxide interface [20,21].

**Figure 2:**
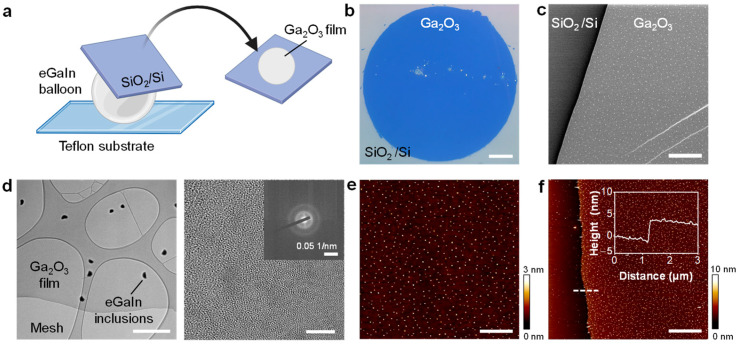
Characterization of the gallium oxide film transferred from an eGaIn balloon. (**a**) Schematic illustration of the transfer process. (**b**) Optical microscope image of the transferred gallium oxide film on a SiO_2_/Si substrate. Scale bar: 50 μm. (**c**) SEM image of the gallium oxide film. Scale bar: 2 µm. (**d**) TEM image (**left**) and HR-TEM image (**right**) with the selective area electron diffraction (SAED) pattern shown in the inset. Scale bars: 500 nm for the TEM image and 5 nm for the HR-TEM image. (**e**) AFM image of the gallium oxide film. Scale bar: 2 μm. (**f**) AFM image of gallium oxide film near the edge, with a height profile along the dotted line displayed in the graph. Scale bar: 4 μm.

The formation of a liquid metal balloon can be explained by three critical factors. First, the applied air pressure must exceed the critical surface stress of the oxide skin to initiate the expansion. Second, the gallium oxide layer demonstrates remarkable elasticity, with an elastic modulus (G_S′_~11–13 N/m) [22] enabling significant stretching without structural failure [23]. Third, the formation of oxide layers effectively reduces the surface tension of eGaIn from 624 mN/m for pristine eGaIn [24] to 356 mN/m for Ga_2_O_3_ [25], similar to how soap reduces the surface tension of water from 72 mN/m to 35 mN/m in soap bubbles. The presence of oxide layers on both the interior and exterior surfaces creates a unique thin-film architecture that stabilizes the balloon. Here, the gallium oxide layer forms rapidly—even a minimal partial pressure of oxygen is sufficient to form a stable gallium oxide-enriched surface layer [26]. Regan et al. reported that an exposure of 180 L (1 L = 10^−6^ Torr partial pressure for one second) creates a complete surface layer, while increased exposure to 800 L and heating up to 300 °C did not significantly increase oxide thickness [21]. After the initial formation of gallium oxide (~0.5–3 nm), further thickening oxide proceeds very slowly and follows logarithmic behavior, even with extended oxygen exposure. The measured exfoliated oxide thickness of ~4 nm (Figure 2f) aligns with these previous studies [4,21].

The translucent gallium oxide layer was transferred from the liquid metal balloon onto substrates for characterization. The transfer was accomplished by gently touching the balloon with a target substrate, either a SiO_2_/Si wafer or a transmission electron microscopy (TEM) grid, as illustrated in Figure 2a. Using our liquid metal balloon approach, we successfully exfoliated circular gallium oxide thin films with diameters of up to 300 μm. Optical microscopy of the transferred gallium oxide film exhibited an evenly blue color, suggesting the uniform thickness of the film (Figure 2b). Scanning electron microscopy (SEM) of the film also showed a smooth surface without any large residues of eGaIn (Figure 2c). The elemental composition of the film, as estimated by energy-dispersive X-ray spectroscopy (EDX), was ~40 at.% Ga and 60 at.% O, supporting that the film was indeed Ga_2_O_3_. Although it was not clear in the optical and scanning electron microscopy, a TEM image revealed many eGaIn inclusions within the Ga_2_O_3_ film (Figure 2d, left) that could not be completely separated from the gallium oxide during inflation. The high-resolution TEM image and selective area electron diffraction (SAED) pattern confirmed the amorphous structure of the gallium oxide film (Figure 2d, right). High-magnification EDX elemental mapping of the Ga_2_O_3_ film demonstrated a uniform distribution of Ga elements throughout the film, along with eGaIn inclusions showing both Ga and In elemental distributions (Figure S1). The height of these eGaIn inclusions was further measured using an AFM height profile, revealing that the eGaIn inclusions were approximately 3–5 nm in height (Figure S2).

Further characterization using atomic force microscopy (AFM) showed the surface morphology of the film with a root-mean-square surface roughness of 0.439 nm (Figure 2e). The height profile across the interface between silicon substrate and the gallium oxide layer revealed the Ga_2_O_3_ film thickness to be approximately 4 nm (Figure 2f, inset). The combination of an amorphous structure, ultrathin thickness, and uniform surface makes this gallium oxide film a unique 2D material.

**Figure 3:**
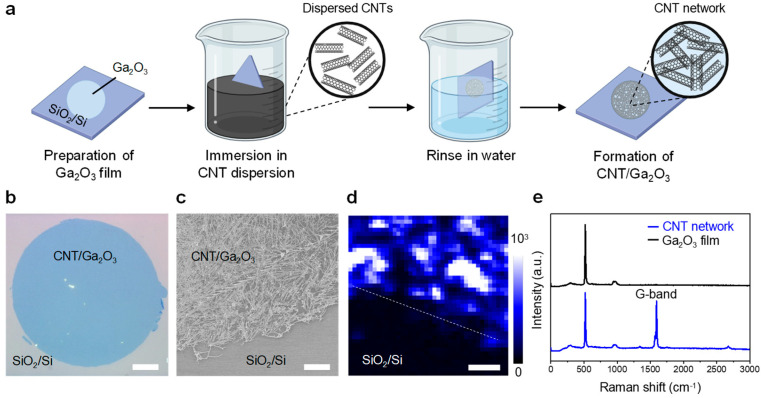
Selective deposition of CNT network on a gallium oxide film. (**a**) Schematic illustration showing the deposition of CNTs dispersed in aqueous solution onto the Ga_2_O_3_ film, which leads to the formation of a random network of CNTs on the Ga_2_O_3_ film. (**b**) Optical image of the CNT/Ga_2_O_3_ film. Scale bar: 50 μm. (**c**) SEM image of the CNT/Ga_2_O_3_ film collected from the boundary between the CNT/Ga_2_O_3_ region and the silicon substrate. Scale bar: 2 μm. (**d**) Raman map of the G-band (1585–1600 cm^−1^) from the boundary between the CNT/Ga_2_O_3_ region and the silicon substrate. Scale bar: 4 μm. (**e**) Raman spectra of the Ga_2_O_3_ film, derived from an eGaIn balloon, after immediate immersion (blue) or delayed immersion (black) in the CNT dispersion.

We found that the gallium oxide layer exfoliated from the eGaIn balloon selectively binds to CNTs dispersed in water, enabling the formation of a percolating network of CNTs for further application in electronics. Here, our choice of CNTs over other nanomaterials was motivated by their strong affinity for oxide layers, as previously observed in studies on CNT/eGaIn composites [27]. Figure 3a illustrates the step-by-step procedure for the selective deposition of CNTs on Ga_2_O_3_. The gallium oxide film freshly transferred from the balloon was immediately immersed in a CNT dispersion for 5 min, during which the CNTs were selectively adsorbed only on the Ga_2_O_3_ layer to form a CNT/Ga_2_O_3_ film. The substrate was then rinsed with water, followed by gentle drying with a nitrogen gun. The successful deposition of CNTs selectively on fresh gallium oxide was confirmed by SEM imaging and Raman spectroscopy. An optical image of the CNT/Ga_2_O_3_ film is shown in Figure 3b. An SEM image of the edge of the CNT/Ga_2_O_3_ region confirmed the formation of a CNT network selectively on the gallium oxide region (Figure 3c). The selective deposition of CNTs was further verified by mapping the G-band (1585–1600 cm^−1^) of CNTs (Figure 3d) obtained from individual Raman spectra of the CNT/Ga_2_O_3_ film (Figure 3e, blue). The resulting percolated CNT network on the gallium oxide film demonstrates promising potential for device applications.

It is worth noting that the CNT adsorption is sensitive to the freshness of the transferred gallium oxide. As described above, the freshly exfoliated gallium oxide film can be used to form a dense network of CNTs. However, gallium oxide films that were kept in the air for a few hours before being immersed in the CNT dispersion did not bind to the dispersed CNTs, as evidenced by SEM images (Figure S3). The Raman spectrum also showed no sign of CNT adsorption on such gallium oxide, which was immersed in the CNT dispersion with a delay (Figure 3e, black).

**Figure 4:**
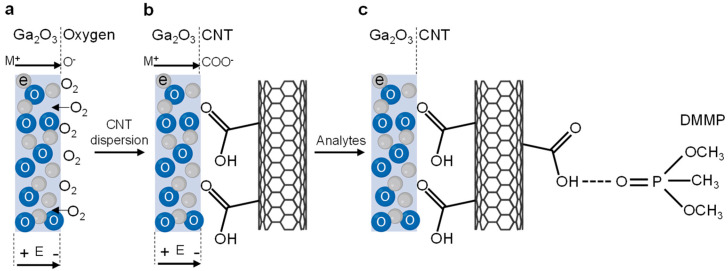
Schematic illustration showing the mechanism of CNT binding on gallium oxide. (**a**) The Mott potential is generated by the adsorption of oxygen molecules during initial gallium oxidation, where e^−^ and M^+^ represent an electron and a metal cation, respectively. (**b**) When the oxygen environment is replaced by a CNT suspension, negatively charged carboxyl groups on the CNTs are attracted to the positively charged gallium metal cations, enabling selective CNT deposition. (**c**) Upon exposure to DMMP vapor, a hydrogen bond forms between the P=O groups of DMMP and carboxylated CNTs, facilitating electron transfer from DMMP to p-doped CNTs, resulting in increased resistance of the CNT network.

The selective self-assembly of CNTs on gallium oxide can be explained by the Cabrera–Mott oxidation model [28,29], as illustrated in Figure 4. During the initial gallium oxidation, oxygen molecules adsorb and ionize at the surface, generating an electrostatic potential (Mott potential, E) at both the oxidizer–oxide and metal–oxide interfaces (Figure 4a). This potential creates an electric field that facilitates metal ion (M^+^) diffusion, promoting oxide growth until it reaches a diffusion limit [30]. When the oxygen environment is replaced by a CNT suspension, negatively charged carboxyl groups on the CNTs are attracted to the positively charged gallium metal ions at the surface, enabling selective deposition (Figure 4b). Recent studies have shown that gallium oxidation is self-limiting and time-dependent [31]. The oxide thickness increases rapidly in the first 60 min but significantly slows down between 60 and 240 min, indicating that the initial rapid oxidation period is crucial for CNT assembly on the oxide. The thickening oxide layer reduces the electric field strength as the Mott potential distributes across the increased thickness, eventually diminishing electron tunneling and ion diffusion processes. This explains why delayed immersion of the Ga_2_O_3_ film in a CNT suspension results in no CNT deposition.

**Figure 5:**
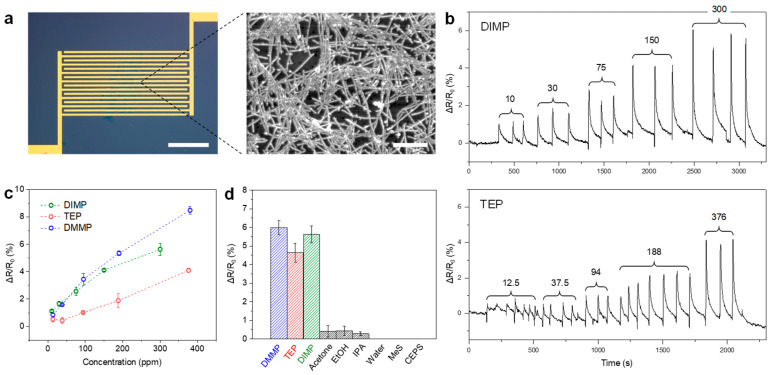
CNT/Ga_2_O_3_-based chemiresistive gas sensor. (**a**) Optical image of the gas sensor fabricated by patterning interdigitated electrodes on a CNT/Ga_2_O_3_ layer, with an SEM image of CNTs shown on the right. Scale bars: 200 μm for the optical image and 1 μm for the SEM image. (**b**) Sensor responses to DIMP (**top**) and TEP (**bottom**) vapor at various concentrations (ppm) labeled on top. (**c**) Calibration curves showing the sensor responses vs. analyte concentration for DIMP (green), DMMP (blue), and TEP (red). (**d**) Comparison of sensor responses to the analytes and common solvents at 300 ppm.

The selective deposition of CNTs on gallium oxide enables the fabrication of electronic devices, such as sensors, which benefit from the high sensitivity of CNTs to molecular adsorption due to their one-dimensional electronic structure and extremely high surface-to-volume ratio. Here, we demonstrate a CNT/Ga_2_O_3_-based chemiresistor for the detection of simulants of chemical warfare agents (CWAs): diisopropyl methylphosphonate (DIMP), dimethyl methylphosphonate (DMMP), and triethyl phosphate (TEP). The sensor was fabricated by patterning interdigital electrodes on the CNT/Ga_2_O_3_ film, as shown in the optical image (Figure 5a, left). An SEM image shows a percolating network of CNTs bridging the gap (Figure 5a, right). The baseline resistance of the sensors, prior to exposure to the analytes, was ~600 Ω. Upon exposure to the CWA simulants, the sensor resistance increased, and the responses were reversible over a wide range of concentrations, as clearly shown in the responses to DIMP (Figure 5b, top), TEP (Figure 5b, bottom), and DMMP (Figure S4). These results were used to generate calibration curves for the three analytes (Figure 5c). The limits of detection (LOD) were 13 ppb for DIMP, 28 ppb for DMMP, and 53 ppb for TEP, which were estimated by extrapolating the response at the lowest tested concentration down to three times the noise level. In terms of LODs, our CNT/Ga_2_O_3_ chemiresistors outperform paper-based inkjet-printed CNT chemiresistors (3 ppm for DMMP) [32] and CNT-based chemicapacitors (700 ppb for DMMP) [33], and demonstrate LODs approaching those of graphene chemiresistors (5 ppb for DMMP) [34]. A control device containing only the Ga_2_O_3_ film without CNTs showed high resistance (>400 kΩ) and no response to any tested vapors (DMMP, DIMP, TEP, acetone, ethanol, IPA, and water; Figure S5), confirming that the resistive responses originated solely from analyte adsorption on CNTs. Notably, testing CNTs alone was not feasible as they cannot be directly deposited on SiO_2_/Si substrates, highlighting the crucial role of the Ga_2_O_3_ film as a support material that enables successful CNT deposition. The sensor demonstrated selectivity toward organophosphate-based CWA simulants (DIMP, DMMP, and TEP) by showing no response to saturated vapors of methyl salicylate (MeS) and 2-chloroethyl phenyl sulfide (CEPS) (Figure S6). Furthermore, comparison of the responses to the CWA simulants, common organic solvents, and humidified air at the same concentration of 300 ppm (Figure 5d) showed higher sensitivity toward the CWA simulants. The sensor’s selectivity and sensitivity can be further enhanced by coating CNTs with various selective receptors that bind selectively to target analytes. Further, improvements could be achieved by forming sensor arrays coated with different receptors and analyzing their response patterns from sensor arrays.

The sensing mechanism of the device involves an interfacial charge transfer network, where P=O groups in DMMP interact with carboxylated CNTs via hydrogen bonding, as illustrated in Figure 4c. When DMMP molecules adsorb on the sensor surface, an electron transfer occurs from DMMP to p-doped CNTs [35]. This electron donation pathway, facilitated by the carboxyl groups on the CNT surface, leads to a reduction in available charge carriers (holes) in the CNTs. Consequently, the electrical resistance of the CNT network increases, providing a measurable signal for DMMP detection.

## 3. Materials and Methods

### 3.1. Materials and Reagents

All chemicals and reagents were used without any additional purification process. The eGaIn alloy, composed of 75.5% Ga and 24.5% In by weight (99.99% purity), was purchased from Sigma-Aldrich (St. Louis, MO, USA). DMMP, DIMP, and TEP were also obtained from Sigma-Aldrich. Acetone and isopropanol were purchased from SK chemicals. Ethanol was sourced from Daejung chemicals. Deionized water was prepared using a Milli-Q water purification system (Millipore, Darmstadt, Germany). A 1 mL gastight syringe (model 1001TLL, Hamilton, Reno, NV, USA) and a blunt-tip needle (34-gauge, 67 mm long) were purchased from Hamilton and Sigma-Aldrich, respectively.

### 3.2. Dispersion of CNTs

Arc-discharge single-walled carbon nanotubes purified by nitric acid (P3-SWNT, Carbon Solutions, Riverside, CA, USA) were dispersed in deionized water at a concentration of 0.5 mg/mL. The CNT dispersion was prepared by mild sonication in an ultrasonic bath for 1 h, followed by tip sonication at 5 W for an additional hour. The dispersion was centrifuged at 14,000 rpm for 90 min to remove large CNT bundles and aggregates. The supernatant, containing individually dispersed CNTs and small bundles, was collected for use in subsequent procedures.

### 3.3. Formation of CNT Network on Gallium Oxide Film

The translucent part of the eGaIn balloon, representing a thin film of gallium oxide, was gently transferred onto a SiO_2_/Si substrate. Any residual eGaIn was removed by immersing the transferred film in ethanol, followed by thorough rinsing with ethanol. The substrate was then immersed in a CNT suspension for 5 min to enable the self-assembly of a CNT network on the gallium oxide. Excessive CNTs were removed by rinsing with water and gentle drying with a nitrogen gun.

### 3.4. Fabrication and Testing of CNT/Ga_2_O_3_-Based Gas Sensors

Electrodes were patterned on the CNT network by conventional photolithography. Briefly, a positive photoresist (AZ5214E) layer was spin-coated onto the SiO_2_/Si substrate containing the CNT/Ga_2_O_3_ film at 4000 rpm for 30 s. The photoresist was soft baked at 105 °C for 90 s, exposed to UV light (365 nm) at 100 mJ/cm^2^ through a chrome mask, and developed in an AZ 500 MIF developer (AZ Electronic Materials) for 1 min. Finally, electrodes were patterned by depositing Cr (3 nm) and Au (70 nm) films and lifting off the metals in acetone to complete the fabrication of CNT chemiresistors. The sensor response was tested by manually flowing 60 mL of analyte vapor onto the sensor for 10 s while recording its resistance using a semiconductor parameter analyzer (4200A-SCS, Keithley, Cleveland, OH, USA). The desired analyte concentration was achieved by diluting saturated analyte vapor with air. All measurements were conducted at 20 °C and 41% relative humidity.

## 4. Conclusions

This study demonstrates the exfoliation of ultrathin gallium oxide (Ga_2_O_3_) layers from liquid metal balloons and their ability to selectively adsorb CNTs dispersed in water, forming dense, percolating networks suitable for device fabrication. As an example, a CNT/Ga_2_O_3_ composite was used to create a chemiresistive gas sensor capable of detecting simulants of chemical warfare agents (CWAs) with reversible responses, high sensitivity, and low limits of detection. Despite these promising results, the current method is limited by the small size of the liquid metal droplet and the translucent area of the Ga_2_O_3_ layer, restricting the preparation of large-area films. Future efforts should focus on scaling up the exfoliation of Ga_2_O_3_ film and exploring the functionalization of CNTs to expand the range of applications in sensing and electronic devices.

## Data Availability

Data are contained within the article and Supplementary Materials.

## Supplementary Materials

The following supporting information can be downloaded at https://www.mdpi.com/article/10.3390/molecules29245894/s1, Figure S1: High-magnification EDX elemental mapping of Ga_2_O_3_ thin film; Figure S2: AFM height profile of eGaIn inclusions in film; Figure S3: Time-dependent selective deposition of CNT network on Ga_2_O_3_ film; Figure S4: Sensor responses to DMMP; Figure S5: Responses of Ga_2_O_3_ film to analytes; Figure S6: Responses of CNT/Ga_2_O_3_ to MeS and CEPS.
