# Supplementary material for: A Liquid Metal Balloon for the Exfoliation of an Ultrathin and Uniform Gallium Oxide Layer
Source: Molecules. 2024 Dec 13;29(24):5894. doi: 10.3390/molecules29245894 (PMC11677224; doi:10.3390/molecules29245894)
Supplement: Supplementary file 1 [file molecules-29-05894-s001.zip › molecules-3351578-supplementary.pdf]

Supplementary Information

A Liquid Metal Balloon for the Exfoliation of Ultrathin and Uniform Gallium Oxide Layer

Anar Zhexembekova, Seongyeop Lim, Cheongha Lee, Yun-Tae Kim, and Chang Young Lee*

School of Energy and Chemical Engineering, Graduate School of Carbon Neutrality, Ulsan National Institute of Science and Technology (UNIST), Ulsan 44919, Republic of Korea

*Correspondence: cylee@unist.ac.kr

This PDF file includes Supporting Figures S1-S6.

Supporting Figures

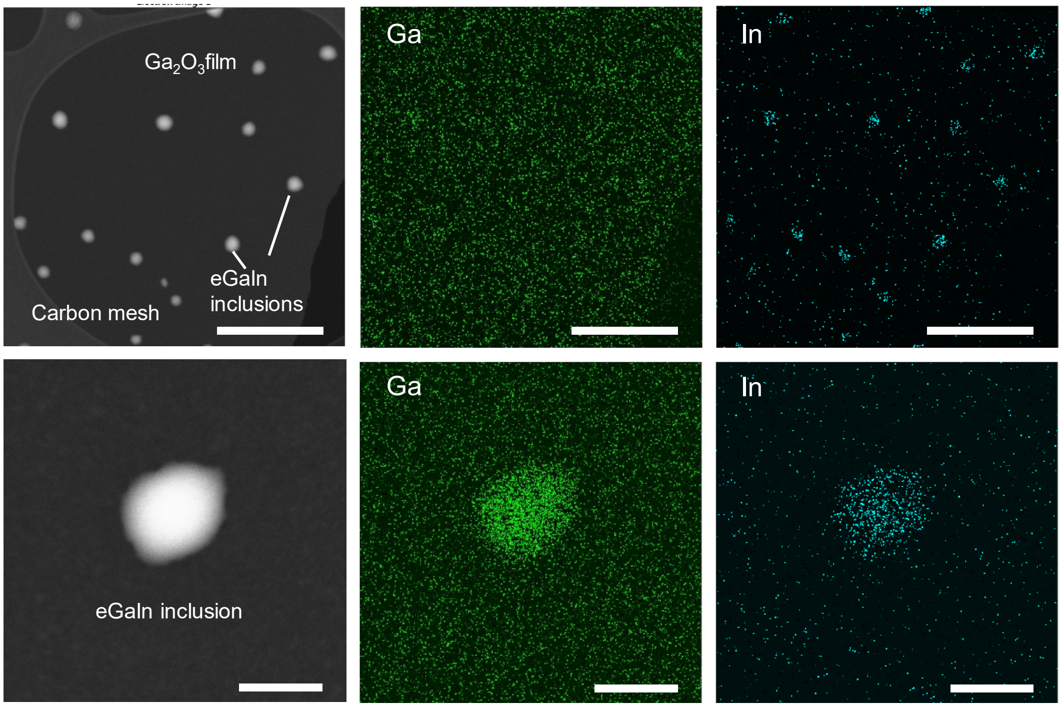

Figure S1. High-magnification EDX elemental mapping of the Ga_2O_3 thin film. The images demonstrate the uniform distribution of Ga throughout the film, along with eGaIn inclusions consisting of both Ga and In. Scale bars: 500 nm.

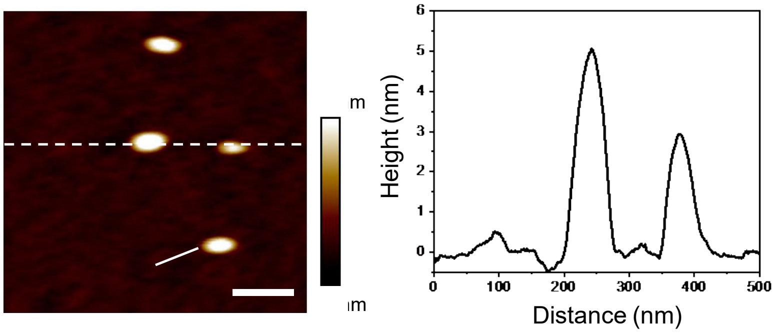

Figure S2. AFM height profile along the white dotted line in the AFM image. The profile reveals that the nano-sized inclusions in the oxide thin film have heights of approximately 3–5 nm. Scale bar: 100 nm.

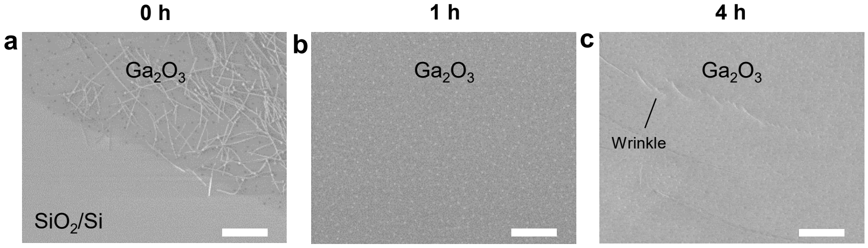

Figure S3. Time dependence of CNT deposition on the Ga_2O_3 film. SEM images of the Ga_2O_3 film on a silicon substrate immersed in the CNT dispersion: (a) immediately (0 hours) after transfer from the eGaIn balloon, (b) 1 hour after transfer, and (c) 4 hours after transfer. Scale bars: 2 μm.

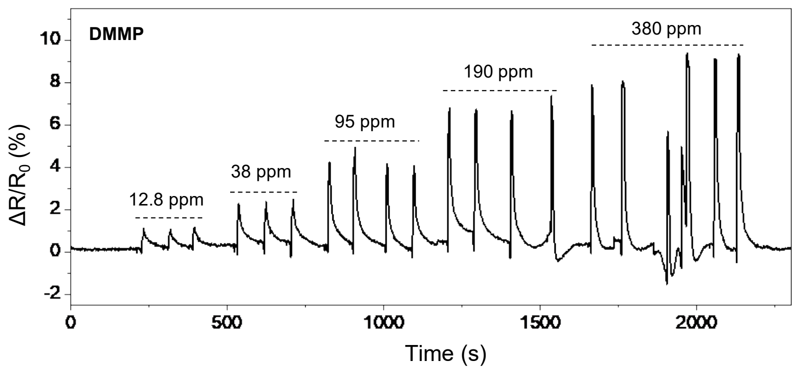

Figure S4. Sensor responses to DMMP vapor at various concentrations (ppm) labeled on top.

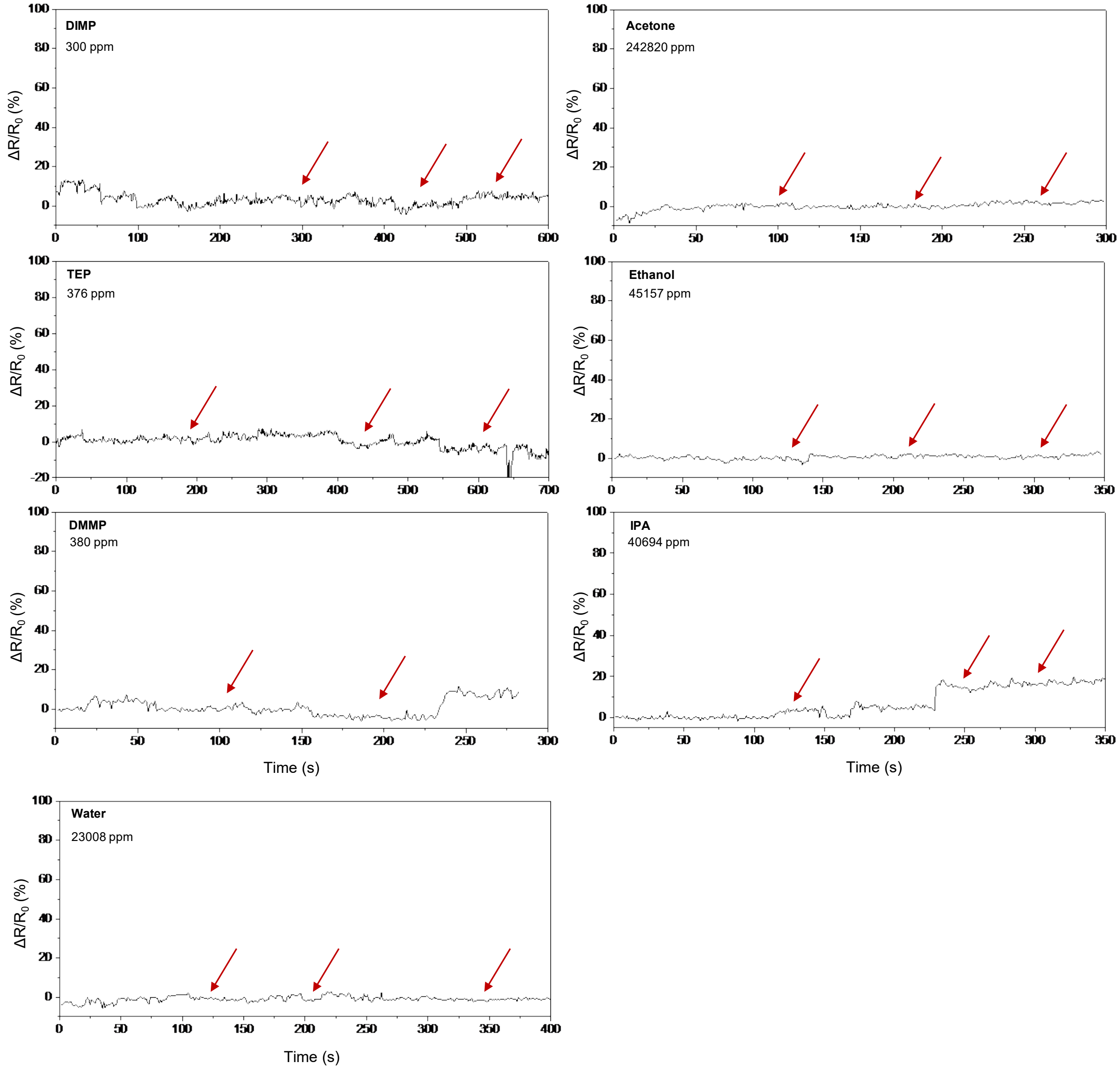

Figure S5. Responses of Ga_2O_3 films to various analytes: DIMP (300 ppm), TEP (376 ppm), DMMP (380 ppm), acetone (242,820 ppm), ethanol (45,157 ppm), IPA (40,694 ppm) and water (23,008 ppm). Red arrows indicate where the films were exposed to the analytes.

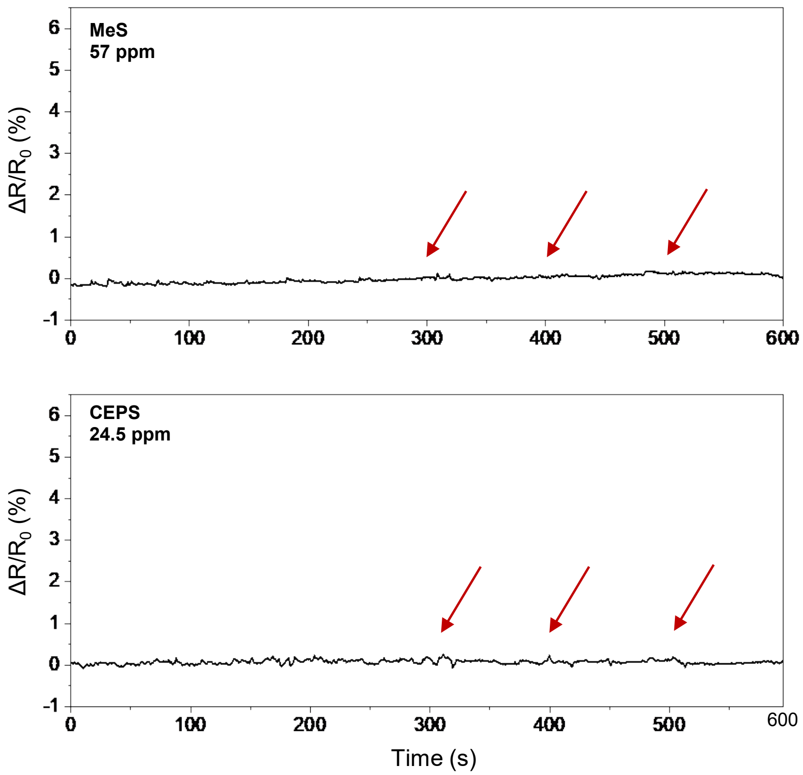

Figure S6. Sensor responses to saturated vapors of MeS (top) and CEPS (bottom) with the concentrations labeled on top. Red arrows indicate vapor injection times.
